# Effect of Polymer Viscosity and Polymerization Kinetics on the Electrical Response of Carbon Nanotube Yarn/Vinyl Ester Monofilament Composites

**DOI:** 10.3390/polym13050783

**Published:** 2021-03-04

**Authors:** Omar Rodríguez-Uicab, Ian Guay, Jandro L. Abot, Francis Avilés

**Affiliations:** 1 Department of Mechanical Engineering, The Catholic University of America, Washington, DC 20064, USA; rodriguezuicab@cua.edu (O.R.-U.); 37guay@cua.edu (I.G.); abot@cua.edu (J.L.A.); 2 Centro de Investigación Científica de Yucatán A.C., Unidad de Materiales, Calle 43 No. 130 × 32 y 34, Col. Chuburná de Hidalgo, C.P. 97205 Mérida, Yucatán, Mexico

**Keywords:** carbon nanotube yarn, electrical resistance, curing, vinyl ester, initiator, viscosity

## Abstract

The effect of polymerization kinetics and resin viscosity on the electrical response of a single carbon nanotube yarn (CNTY) embedded in a vinyl ester resin (VER) during polymerization was investigated. To analyze the effect of the polymerization kinetics, the concentration of initiator (methyl ethyl ketone peroxide) was varied at three levels, 0.6, 0.9, and 1.2 wt.%. Styrene monomer was added to VER, to reduce the polymer viscosity and to determine its effect on the electrical response of the CNTY upon resin wetting and infiltration. Upon wetting and wicking of the CNTY by VER, a transient decrease in the CNTY electrical resistance (ca. −8%) was observed for all initiator concentrations. For longer times, this initial decrease in electrical resistance may become a monotonic decrease (up to ca. −17%) or change its trend, depending on the initiator concentration. A higher concentration of initiator showed faster and more negative electrical resistance changes, which correlate with faster gel times and higher build-up of residual stresses. An increase in styrene monomer concentration (reduced viscosity) resulted in an upward shift of the electrical resistance to less negative values. Several mechanisms, including wetting, wicking, infiltration, electronic transfer, and shrinkage, are attributed to the complex electrical response of the CNTY upon resin wetting and infiltration.

## 1. Introduction

The goal of carbon nanotube yarn (CNTY) production is to translate the excellent properties of individual carbon nanotubes (CNTs) to this continuous fiber assembly [1]. Due to their hierarchical assembly, CNTYs possess outstanding electro-mechanical properties, which can be utilized for advanced sensory applications and mechanical reinforcement [2,3,4,5]. The success in retaining most of the physical properties after the hierarchical (from nano- to micro-scale) assembly of the yarn can be correlated back to the morphology, architecture, and density (porosity) of the CNTY, which in turn is a function of the synthesis method [6]. Regarding CNTYs dependence of electrical resistance with strain, the piezoresistive response of the yarn is driven by changes in its conductive network. This is a function of contact points and tunneling due to CNT and CNT bundles/fibrils proximities [7,8]. The dependence of mechanical, thermal, and chemical enviroments on the electrical response of CNTYs, combined with their tailorable structure, flexibility, and high surface area, makes them excellent candidates for sensory applications [3,9,10,11]. Research regarding the implementation of CNTYs into viable applications has addressed the study of stand-alone and embedded fibers as reinforcements, actuators, energy-storage applications, light emission and sensors for damage, strain, chemical, bio, and wearable technology [12,13,14,15,16,17,18,19,20,21,22,23,24,25]. One of the key aspects of this electrical sensitivity, which controls the response of the yarn, is its porosity. CNTYs are highly porous materials, with typical porosities ranging from 40% to 90% [26,27,28]. This high porosity allows infiltration of moisture, liquids, and chemical species, which in turn changes the internal morphology and microstructure of the yarn, changing its electrical conductivity [3,29,30]. Therefore, the electrical conductivity of CNTYs has been shown to be sensitive to the ingress or presence of surrounding liquids [30,31]. It has been shown that the electrical resistance of CNTYs responds differently if the CNTY is set in direct contact with polar liquids/solvents, non-polar liquids, or epoxy/vinyl ester resins [3,29,30]. Ingress of low viscosity liquids and solvents into CNTYs has experimentally shown increments in the longitudinal electrical resistance larger than 10% [3]. Infiltration can separate adjacent bundles and cause fiber swelling, increasing bundles proximity and contact resistance [30]. These elastocapillary effects could also be a factor for the change in electrical resistance upon wetting [3,32]. However, it has also been shown that electrochemical doping of the surface of the CNTY may play a paramount role, depending on the polarity of the solvent [3,29]. Upon immersing CNTYs into liquid thermosetting polymers, it has been found that ingress of the polymer resin into the porous CNTY occurs without the need of pressure, i.e., driven by capillary forces [33,34]. Research has showed small CNTY swelling after infiltration, which suggests that the viscous polymer infiltrates the voids between bundles, but not the voids within the bundles [3,4]. Fernández-Toribio et al. [3] reported the change in electrical resistance during full immersion of a CNTY in a thermosetting polymer containing epoxy vinyl ester resin (VER), methyl ethyl ketone peroxide (initiator), and cobalt octoate (promoter), at a ratio of 100:1.5:0.3 in weight. Upon immersion, a fast decrease in resistance was observed. This drop in electrical resistance was attributed to the presence of highly reactive free radical groups, acting as electron donors, formed during the polymerization of the VER [3]. However, the electrical response may depend on the resin viscosity and concentration of initiator, and several unanswered questions and undeciphered mechanisms still prevail, given the complexity of the phenomenon. Therefore, this study investigates the use of a CNTY as an in situ sensor for monitoring polymer curing, through the electrical response of a single CNTY embedded into a thermosetting resin (VER). Particular emphasis lies on the investigation of the role of the polymerization reaction initiator and the effect of resin viscosity on the electrical response of the CNTY during resin curing.

## 2. Materials and Methods

### 2.1. Materials

**Figure 1:**
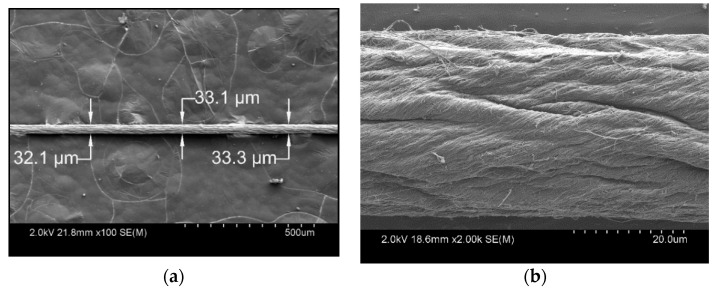
SEM images of a section of carbon nanotube yarn (CNTY): (**a**) ×100 and (**b**) ×2000.

CNTYs were synthesized via chemical vapor deposition at the “Nanoworld Laboratories” of the University of Cincinnati (Cincinnati, OH, USA). The yarns possess a bulk density of ~0.65 g/cm^3^, a measured diameter of ~32.8 µm, and a twist angle (*θ*) of ~30°. Using the density of the constituent multiwall CNTs as 1.76 g/cm^3^ [35], the estimated porosity of the CNTY, using its bulk density, is 0.63. As seen from the scanning electron microscopy (SEM) image in Figure 1, the continuous CNTY comprises thousands of twisted multiwall carbon nanotubes (MWCNTs) and CNT bundles. Commercial epoxy vinyl ester resin (VER), Derakane Momentum 470-300, from Ashland Global Specialty Chemicals (Covington, KY, USA), was used as the polymer matrix. The resin, as received from the manufacturer, has 33 wt.% styrene content. Methyl ethyl ketone peroxide (MEKP) and cobalt naphthenate (CoNap) were used as the initiator and promoter, respectively. The concentration of CoNap was fixed at 0.6 wt.% for all experiments, and 0.9 wt.% was used as the nominal concentration for MEKP. Dedicated experiments to investigate the effect of the initiator concentration on the electrical response of the CNTY were conducted by varying the concentration of MEKP by ±0.3 wt.% with respect to the nominal concentration, to obtain lower (0.6 wt.%) and higher (1.2 wt.%) MEKP concentrations. For selected experiments, styrene monomer, “FibreGlast #70 pure styrene thinner” (Brookville, OH, USA), was added to the neat VER, to alter (decrease) the polymer viscosity, in order to determine its effect on infiltration and the resulting electrical response of the CNTY.

### 2.2. Specimen Preparation and Setup

**Figure 2:**
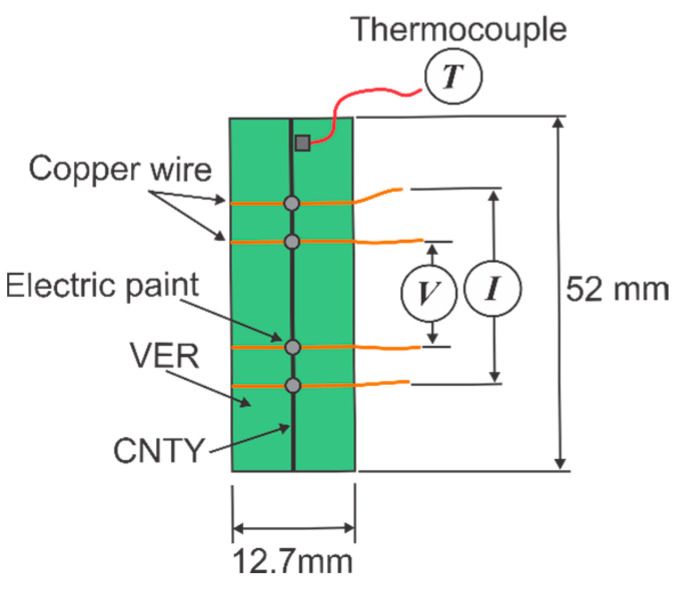
Schematic depicting the experimental setup of in situ electrical measurements and dimensions.

To measure the (direct current) electrical resistance response of the CNTY during VER curing, a 52 mm—long, 12.7 mm—wide, and 2.5 mm—deep silicon mold was constructed, and the CNTY was placed centered inside such a mold (Figure 2). The CNTY was pre-stretched by hanging a constant weight of 0.712 mg mass from the end of the CNTY, which was equivalent to a 0.698 mN force. Assuming a solid CNTY cross section of 8.45 × 10^−10^ m^2^, this corresponds to a pre-stress of 826 kPa, which is ~1.6% of the yarn strength, according to the strength reported for tensile tested as-spun CNTYs with twist angle ranging from 10° to 25° [28]. Four AWG 426-DFV copper wire electrodes (Vishay MicroMeasurements, Wendell, NC, USA) were inserted spaced as shown in Figure 2, centered within the length and at the mid-depth of the silicon mold, spanning its width and overhanging at the other end of the mold. A single pre-stressed CNTY was then positioned transversely to the four wires and lying on top of them, and its end was adhered to the mold with tape and commercial Scotch-Weld LO1000 adhesive (3M, St. Paul, MN, USA). To ensure Ohmic contact between the CNTY and the copper electrodes, electric paint (Bare Conductive, London, UK) was applied to each of the four contact points. All experiments were conducted with the CNTY placed within the silicon mold, and electrical measurements were taken in situ, prior, during, and after VER pouring. The volume of the resin mixture within the silicon mold used in these experiments was ~1.65 mL. The four-point probe Kelvin resistance technique was used to calculate electrical resistance (R), by measuring the voltage drop (V) between the inner electrodes, under an applied constant current (I), as depicted in Figure 2.

Electrical resistance (*R*) was measured using a PXI-4072 digital multimeter card from NI (Austin, TX, USA), mounted into a PXI-1033 chassis. An NI-9219 universal analog input module and a K-type thermocouple were used to simultaneously measure temperature (*T*) and concurrently correlate it to the measured R of the CNTY. NI Signal Express 2015 software was used for simultaneously acquiring both electrical and thermal data at 1 data/s. Each experiment began by simultaneously recording *R* and *T* for a brief period (17 min) prior to the introduction of VER, in order to capture the initial stabilization stage of *R* before resin pouring. The VER polymer mixture, previously mixed with promoter and initiator, was then poured into the mold. All electrical tests were conducted at room temperature (~25 °C) and their duration ranged between 59 min and 383 min, depending on the experiment.

The effect of the curing kinetics and the effect of resin viscosity were investigated. The effect of the curing kinetics was investigated by varying the amount of initiator (MEKP) according to Table 1. Reaction mixtures consisted of “low” (0.6 wt.%), “nominal” (0.9 wt.%), and “high” (1.2 wt.%) initiator (MEKP) concentrations. A fixed promoter (CoNap) concentration of 0.6 wt.% was added to all three mixtures. A total mass (VER+initiator+promoter) of 30 g was maintained for each mixture. Three replicate tests were produced for each initiator experiment, and representative experiments are shown.

The effect of resin viscosity was investigated by varying the styrene concentration according to Table 2. The as-received VER contains 33 wt.% styrene monomer. This concentration was denoted as the one producing a “nominal” viscosity. Both viscosity mixtures consisted of the same concentrations of Derakane Momentum 470-300 (98.5 wt.%), CoNap (0.6 wt.%), and MEKP (0.9 wt.%) as the nominal initiator mixture. A low viscosity mixture was created by adding 3.28 g of styrene monomer to 29.6 g of resin. This produced a 7% increase in styrene monomer concentration (40 wt.% styrene monomer) with respect to the nominal viscosity mixture. The initiator and promoter concentrations were calculated after the addition of the styrene monomer, as shown in Table 2. Three replicate tests were produced for each viscosity experiment.

Gel times and peak exotherms (peak value of temperature during the VER curing reaction) were also measured, to correlate with the electrical measurements. However, since the exotherm is a function of the resin volume, the ~1.65 mL VER mixture contained in the silicon mold yielded only modest temperature changes. To increase the temperature changes during the curing reaction, supplemental gel time and exothermic experiments were conducted using disposable polypropylene cups containing 14 mL of resin volume, which is ~8.5 times more than the volume of the coupon used for electrical measurements in Figure 2. Thus, exotherms and gel times reported herein were measured on 14 mL resin containers, following identical conditions and concentrations to those used for the experiments depicted in Figure 2. Results show that both temperature changes match, but temperatures changes measured in the ~1.65 mL coupons were far smaller.

### 2.3. Rheometry and Gel Time Measurements

Gel times were measured in plastic containers of 74 mL, using 14 mL of VER. The gel times were measured, using a small wooden spatula inserted perpendicular to the liquid material every 15 s. The exothermic temperature was measured using a thermocouple connected to the data acquisition system. The gel time and peak exothermic temperature were determined according to the ASTM standard D2471 [36]. Derakane Momentum 470-300 resin and CoNap (0.6 wt.%) were initially mixed. Once the desired amount of MEKP was added, the recording of gel time started, and all constituents were then mixed for 3 min. The liquid center of the reacting mass was probed with a thin wooden spatula every 15 s, until there was a noticeable change in the polymer viscosity. The frequency of sampling was then increased to every 20 s, until the reacting mixture no longer adhered to the end of a clean probe.

Rheometry experiments were conducted, using an ER 2000 rheometer of TA Instruments (New Castle, DE, USA) using a parallel plates accessory with 500 µm gap, with a heating rate of 5 °C/min, from 30 to 150 °C, applying a 0.5 µNm torque. To obtain reproducibility, three replicates of each polymer were analyzed.

### 2.4. Electron Microscopy 

Scanning electron microscopy (SEM) was used to investigate the microstructure of the CNTY and the possible infiltration effects. This was conducted, using a Hitachi SU-70 Schottky field emission SEM gun (Marunouchi, Tokyo, Japan). 

## 3. Results

### 3.1. Curing Characterization of Vinyl Ester Resin

**Figure 3:**
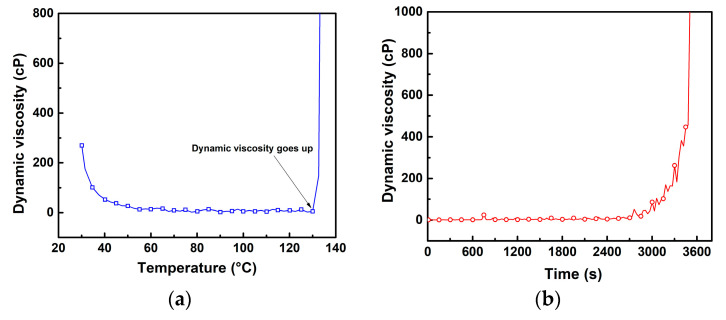
Dynamic viscosity of Derakane Momentum 470-300 resin: (**a**) uncured resin as a function of temperature and (**b**) during curing as a function of time.

Figure 3 depicts rheometry measurements of the Derakane Momentum 470-300 resin (VER) as a function of increasing temperature and time. The VER specimen shown in Figure 3a comprises only the viscous VER in liquid state, with no initiator or promoter added. At 30 °C, the average dynamic viscosity was measured as ~289 cP. The dynamic viscosity given by the manufacturer is 365 cP at 25 °C [37]. The viscosity quickly decreased with increased temperature, reaching 13.6 cP at 60 °C. For temperatures higher than 60 °C, the viscosity does not change much. However, slightly above 130 °C, the viscosity quickly increases to values higher than 800 cP, indicating the beginning of the polymerization process. Figure 3b shows the viscosity as a function of time during curing of a VER mixture (VER+ initiator +promoter). In this figure, the viscosity does not change much during the first ~2700 s (~45 min). However, after this point, the viscosity increases to higher values indicating the beginning of the polymerization process. This curing process occurs through free radical polymerization, which proceeds via a chain growth mechanism [38,39,40,41]. In this process, three types of reactions occur involving radicals, viz initiation, propagation, and termination [38,39,40,41]. From the addition of an initiator to a non-radical species (monomer resin), pairs of free radicals are readily generated from individual initiator molecules [39]. During this reaction, the radical center (electron) transfers from the free radical to the end of the monomer molecule, and thus, the unpaired electron remains unchanged [38,39]. This produces an initial chain that effectively maintains the functionality of the initial free radical (initiation). This free radical continues to grow in this manner, successively bonding to monomer molecules at an increasing rate (propagation). With increasing monomer conversion, the temperature of the reaction system increases, due to the exothermic nature of polymerization [38,39,40,41]. This arises from the exothermic conversion of π-bonds in monomer molecules into σ-bonds in the polymer [38]. Ultimately, the growth of the free radical chain is terminated, which occurs through the combination of free radical chains or disproportionation [38,39,40,41]. 

**Figure 4:**
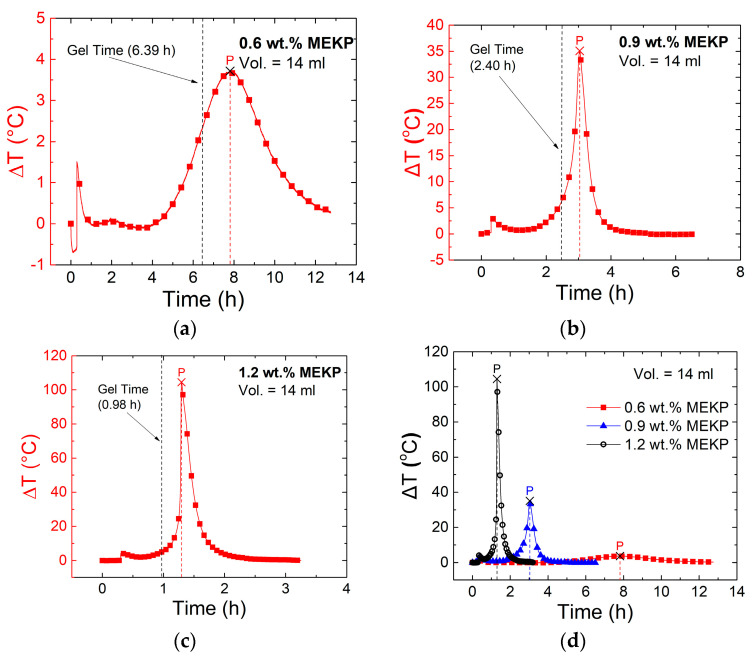
VER thermograms (14 mL) during curing for different MEKP concentrations: (**a**) 0.6 wt.%, (**b**) 0.9 wt.%, (**c**) 1.2 wt.%, and (**d**) superposition of all thermograms.

Figure 4 shows the change in temperature (Δ*T* = *T* − *T*_0_, where *T*_0_ = 25 °C) during VER curing as a function of time for the three concentrations of initiator (MEKP) investigated (Figure 4a–c), using the 14 mL cups. 

A summary of the three initiator concentrations is shown in Figure 4d. Peak exothermic temperatures (at point P) are identified in each plot, and corresponding measured gel times are included alongside their respective curves. Gel times (average and one standard deviation from three replicates) and peak exothermic temperatures are summarized in Table 3.

It is seen that gel time decreases with increased initiator concentration, from 6.39 h for 0.6 wt.% of MEKP to 0.98 h for 1.2 wt.% of MEKP. The decrease in gel time is due to the proportion of additional free radicals formed from the increased amount of MEKP, as seen in Table 1. The increase in MEKP concentration generates more free radicals (more chain initiation sites), which effectively increases the rate of monomer conversion, and, in turn, increases the rate of viscosity change [38,39,40,41,42,43]. As seen from Figure 4 and Table 3, the increase in MEKP concentration was concomitant with a greater exothermic reaction. The amount of heat generated in the reaction is a function of the resin volume [44]. Therefore, an example thermogram using the volume of the coupon shown in Figure 2 (1.65 mL) is shown in Supplementary Materials Figure S1, and the corresponding summary using such lower volume is shown in the Supplementary Material, Table S1. 

For the experiments using the 14 mL volume, the change in temperature for the three tests ranged from ~4 to ~105 °C. It is also seen that all gel times preceded the time when the peak exothermic temperature was experienced (Δ*T* peak position in Table 3) and occurred during or right after the onset of temperature rise (see Supplementary Materials Table S1 for 1.65 mL volume). Superimposing the temperature curves of the three concentration experiments (Figure 4d) showed, more clearly, the trends of peak exothermic temperature upon resin gelling with increasing MEKP concentration. 

### 3.2. Effect of Polymerization Kinetics on the Electrical Response of the CNTY

**Figure 5:**
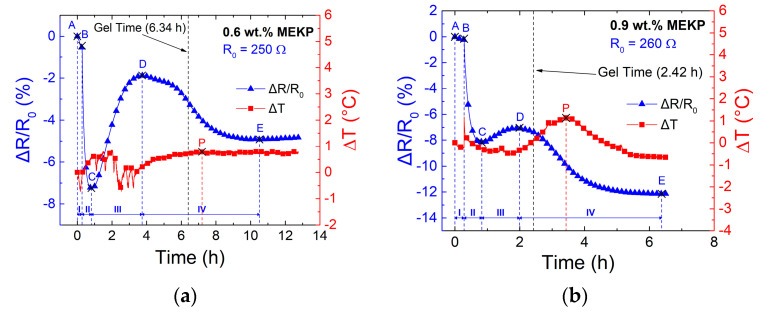

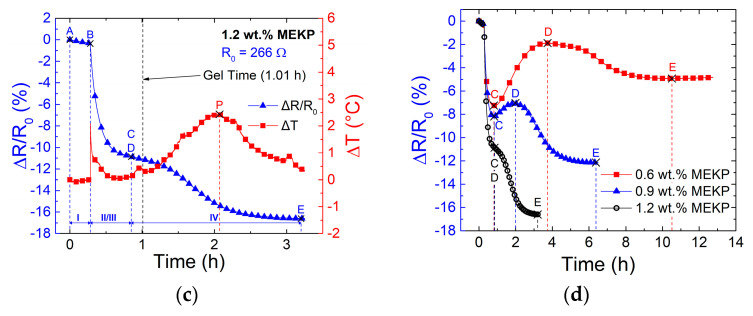
Dynamic evolution of fractional change of electrical resistance and temperature change within the 1.65 mL coupon during VER polymerization for different MEKP concentrations: (**a**) 0.6 wt.%, (**b**) 0.9 wt.%, (**c**) 1.2 wt.%, and (**d**) superposition of the three electrical resistance curves for different MEKP concentrations.

**Figure 6:**
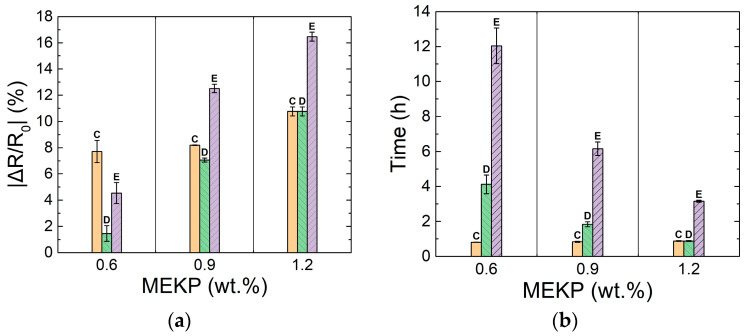
Average values of Δ*R/R*_0_ and their corresponding elapsed times for selected points (C, D, and E). (**a**) Absolute value of Δ*R/R*_0_, (**b**) corresponding elapsed times.

The specimens shown in Figure 2 were used to monitor the fractional change of electrical resistance (Δ*R/R*_0_ = (*R* − *R*_0_)/*R*_0_, where *R*_0_ is the initial electrical resistance before resin pouring, at *t* = 0). These values are shown in the left vertical axis of Figure 5. The right vertical axis of Figure 6 (Δ*T* = *T* − *T*_0_, where *T*_0_ = 25 °C) shows the simultaneous recordings of the thermocouple immersed in the coupon of yarn/resin during the curing experiment (Figure 2), whose resin volume is ~1.65 mL. 

As seen from Figure 5a–d, regardless of the concentration of initiator, all curves show an overall behavior which can be rationalized into four zones, as summarized in Table 4. 

Zone I (A-B) is defined as the stabilization period and corresponds to the initial 17 min where constant temperature and a small constant stress (8.26 × 10^−4^ GPa) is applied to the freestanding (dry) CNTY, prior to the introduction of VER. A slight decrease in electrical resistance is observed for all three concentrations of initiator in this zone, which is attributed to viscoelastic relaxation of the CNTY upon a (small) constant force. As indicated by previous studies [2], the electrical resistance of the CNTY tends to decrease if the yarn is stretched and held at constant force for a few minutes [2]. The curing reaction kinetics of VER depends on the proportions of the initiator (MEKP) and promoter (CoNap). By varying their concentrations and gel time, the extent of shrinkage and peak exothermic temperature are altered [38,39,41]. At point “B” (end of zone I), the reaction mixture was poured into the silicon mold containing the CNTY. This corresponds to the onset of zone II (B-C). The immediate wetting of the CNTY with VER results in a transient drop in electrical resistance in all experiments, until reaching point C. Surface wetting, wicking, and free radicals acting as electron donors, are credited to this response [3,30,33]. This electrochemical phenomenon was physically characterized by VER initially wetting the surface of the CNTY, filling the capillary voids and gaps about its irregular surface, followed by infiltration and wicking between intra-bundle voids [3,30,33]. Resin ingress into textiles and yarns is expected to be driven by capillary penetration, adsorption, and diffusion, which may occur concurrently, driven by capillary forces arising from the large porosity of the CNTY [3,30,33]. Elastocapillary effects due to CNTY swelling has also been mentioned in the literature as a possible contributor [3]. However, positive changes of electrical resistance are expected from this mechanism, and thus, this mechanism was not deemed as a large contributor in this case, at least in the early stages of zone II (early stages of curing), where the free radical electron donors are abundant. Terrones et al. [29] have proved that the increase or decrease in the electrical resistance of the CNTY upon immersion in liquids depends strongly on the polarity of the liquid. 

As the VER wicked further the CNTY, the rate of polymerization increased causing the rate of electrical resistance change to stabilize, presenting a local minimum of Δ*R/R*_0_ at point “C”. At this point, towards the end of zone II (onset of zone III), free radicals become depleted as the resin curing process progresses, due to the increase in monomer conversion [38,39,40,41]. From the beginning of the polymerization process to the gel time, the VER mixture transitions from an electron donor liquid to an insulating material. Therefore, competing mechanisms exist in zone II, which lead to the local stabilization of Δ*R/R*_0_ at point “C”. Point “C” marks the onset of zone III (C-D), which is characterized by the formation of long chains and subsequent cross-linking of these chains. At the beginning of this zone (III), the VER reaction mixture (resin + promoter + initiator) still possesses the ability to flow, so it continues to wick and infiltrate voids within the CNTY. Voids that were once occupied by air are wetted with a highly viscous resin, which is almost depleted of free radical electron donors. As the rate of polymerization increases the resin viscosity increases until the resin nearly gels. Voids up to a certain depth from the yarn’s surface are now occupied by an insulating quasi-solid material with relative permittivity much higher than that of air, which effectively increases the Δ*R/R*_0_ to a local stabilization in point “D”. Point “D” corresponds to the onset of the last zone, zone IV (D-E), where the gelling VER experiences chemical shrinkage (volume contraction), which exerts radial compressive and longitudinal strains on the CNTY [2,45]. 

Chemical shrinkage is believed to reassemble the CNTY’s structure, increasing the density of contact points and decreasing the effective porosity [1,2,35,46,47], hence decreasing Δ*R/R*_0_ in zone IV. The electrical resistance in zone IV decreases until an equilibrium (point E) is reached, which is attributed to the extent of the incurred shrinkage. It can be seen in Figure 5a–c that the temperature change measured within the (~1.65 mL volume) coupon is very small (a few °C) during the experiment. The small peaks of exothermic temperature occur within zone IV but are very small (Δ*T* < 2.7 °C). Therefore, the thermoresistivity of the CNTY itself is expected to be a negligible contributor to Δ*R/R*_0_. From a previous study, the normalized change in electrical resistance (Δ*R/R*_0_) of a CNTY, embedded into a vinyl ester polymer was determined to be linearly proportional to the change in temperature (Δ*T*) within a 25 to 100 °C temperature range [8]. The thermal coefficient of resistance (*β* = (Δ*R/R*_0_)/Δ*T*) of this embedded CNTY (the same kind as the one used herein) was also calculated in such a work as 6.53 × 10^−4^ K^−1^ [8]. Using this temperature coefficient of resistance and a maximum change in temperature of 2.7 °C (~275 K), the decrease in Δ*R/R*_0_ is estimated as ~−0.18%. This is one order of magnitude smaller than what is observed in Figure 5, which proves that thermoresistivity of the CNTY is not the one causing the electrical resistance changes. As seen from the comparison of the three curves in Figure 5d, the dynamic electrical resistance curves for 0.6 and 0.9 wt.% MEKP have a similar form, but with different rates (slopes) and numerical values of Δ*R/R*_0_. As seen from Figure 5c, the point of inflection (point D) nearly coincides with the gel time of the VER, especially as the concentration of initiator increases. More marked transitions are observed in Figure 5a,b for resins with 0.6 wt.% MEKP than for 0.9 wt.%, but the form of the curve (and hence the mechanisms discussed above) prevail. However, for the highest initiator concentration (1.2 wt.%, Figure 5c,d), the trend of Δ*R/R*_0_ is always (monotonically) decreasing, with point “D” basically disappearing or merging with point “C”. This is attributed to the high concentration of free radicals (electron donors), faster curing kinetics, and higher viscosity at the onset of zone III (C-D), causing point “D” for 1.2 wt.% to disappear (or merge with C). Zones II and III for this experiment (1.2 wt.% MEKP) were grouped together for this reason. For this higher concentration of initiator, it is believed that the ingress of VER into the porous yarn was very limited, because of the rapid kinetics of the polymerization phenomenon. The variation in electrical resistance measurements in zone III (and point D) may stem from varying degrees of resin infiltration, where a higher degree of infiltration towards the core of the yarn is expected for the experiment with lower concentration of initiator (0.6 wt.%). In Figure 5d, it is seen that point “D” (onset of zone IV) and point “E” (end of zone IV) shift to the left (less elapsed time, quicker kinetics) as the MEKP concentration increases. The extent of shrinkage depends on the polymerization kinetics [4], and this is obviously more pronounced for the VER with the highest concentration of initiator. Figure 6 summarizes the (absolute) values of the changes in electrical resistance at points “C”, “D”, and “E”, and the corresponding elapsed times when these key points occur, for the investigated concentrations of initiators. 

As seen from Figure 6, the change in electrical resistance at point “C” was similar (~−8%) for 0.6 and 0.9 wt.% initiator, but further increased for 1.2 wt.% (see Figure 6a, where absolute values are plotted). The absolute value of Δ*R*/*R*_0_ increased with the concentration of initiator for points “D” and “E”. All concentrations reached point “C” at about the same time (~50 min, see Figure 6b) but the time needed to reach points “D” and “E” decreased as the concentration of MEKP increased, indicating faster polymerization kinetics. The amount of free radical electron donors formed is a function of the concentration of MEKP, so the highest MEKP concentration (1.2 wt.%) yields the greatest drop (~−11%) in electrical resistance.

### 3.3. Effect of Polymer Viscosity on the Electrical Response of the CNTY

**Figure 7:**
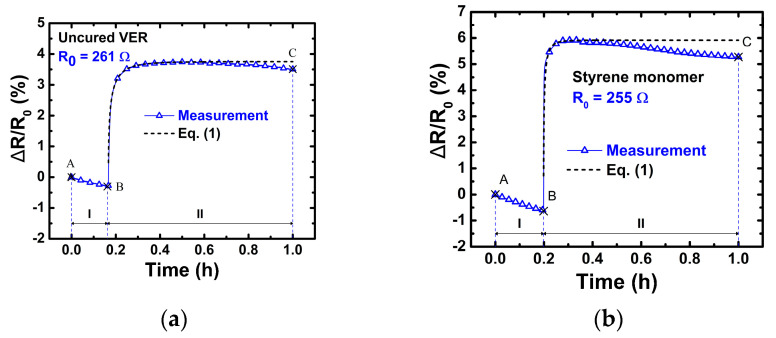
Fractional change of electrical resistance of CNTY versus elapsed time for CNTY immersion into two liquid components of VER: (**a**) uncured VER without initiator or promoter and (**b**) styrene monomer.

To determine the effect of VER viscosity on the electrical response of the CNTY, the adsorption kinetics of the CNTY were initially analyzed by immersing the CNTY in two separate non-polymerizing liquids of differing viscosity, while monitoring the electrical resistance of the CNTY. The two viscous liquids examined are the main constituents of the VER, viz. uncured Derakane Momentum 470–300 (325 cP at 25 °C [37], where neither promoter nor initiator were added) and styrene monomer (0.76 cP at 20 °C [48]). Rheometry analysis of the uncured VER produced dynamic viscosity measurements corresponding to the referenced value in Figure 3. Styrene monomer is known to act as a diluter (“thinner”) of VER in the composites industry, and its viscosity is three orders of magnitude less than that of the VER. Figure 7 shows the electrical response of both experiments. There were no changes in temperature during both experiments, so there were no thermal effects on viscosity. 

The change in electrical resistance of both, the uncured VER (Figure 7a) and styrene monomer (Figure 7b), followed a similar behavior, and the analysis of this behavior was divided into two zones (I and II) delimited by points A, B, and C. The observed behavior corresponds to that of a viscous liquid wicking the porous CNTY [33]. Zone I (A-B) is defined as the stabilization period, prior to the introduction of liquids. A slight decrease in electrical resistance is observed in this period (zone I), which is attributed to a relaxation of the pre-stressed CNTY [2]. At point B (end of zone I), the liquid was poured into the silicon mold (encompassing the yarn). This corresponds to the onset of zone II (B-C), and the initiation of the wetting and wicking mechanisms [33]. The immediate wetting and subsequent wicking of the capillary pores of the CNTY resulted in a rapid increase in electrical resistance. Shortly after, the electrical resistance leveled off (for uncured VER), which suggests the extent of liquid infiltration into the yarn. Sears et al. [49] reported that the packing fraction of CNTs and CNT bundles decreases from the center of the CNTY to its peripheries, so the degree of infiltration is limited to a specific depth. For styrene, however, there is a small decrease in Δ*R*/*R*_0_ towards the end of the experiment, indicating that electronic transfer may still be occurring. This electrical response correlated well with previous studies by Fernández-Toribio et al. [3] of an individual CNTY immersed in non-polymerizing polar and non-polar liquids. In their studies, the changes in electrical resistance were attributed to electrochemical doping of adsorbed liquid molecules on the surface of the porous CNTY [3]. It has been suggested that adsorption of polymers under conditions of low molecular mobility, such as those of a viscous resin into a porous CNTY, can be described by a diffusion process of the form [3,44],

(1) $$\frac{\Delta R\left(t\right)}{R_{0}}={\left(\frac{\Delta R}{R_{0}}\right)}_{\mathrm{Eq}}\;\left(1-e^{-{\left(D\cdot t\right)}^{\gamma }}\right)$$

where (Δ*R/R*_0_)_Eq_ is the equilibrium value of Δ*R/R*_0_ for a sufficiently long time after immersion [3,44]. The exponent *D*·*t* is indicative of a diffusion process, where *D* (cm^2^/s) is the diffusivity per unit cross-section (diffusion coefficient) and *t* is the elapsed time. The results of fitting the diffusion model of Equation (1) to the experimental data of Figure 7 are included in Table 5. Table 5 summarizes the average measured values of viscosities, coefficient of determination (*r*^2^), diffusion coefficient (*D*), exponent factor (*γ*), and equilibrium change in electrical resistance after immersion ((Δ*R/R*_0_)*_Eq_*) for uncured VER and styrene monomer. A very good fit is obtained for the uncured VER, Figure 7a, with an average coefficient of determination (*r*^2^) of 0.97. For the styrene monomer, Figure 7b, given the slight decrease in Δ*R/R*_0_ towards the end of the experiment, *r*^2^ is only 0.47. This is likely because the model is derived for polymers under conditions of low molecular mobility, and the viscosity of the styrene is very low (see Table 4), which may not exactly fulfill this assumption. Electronic exchange may still be occurring for the styrene/CNTY after 1 h. The average exponent (*γ*) is 0.66 for uncured VER and 0.63 for styrene monomer. According to Douglas et al. [44] when the exponent *γ* = 0.5, this model characteristically describes the physical processes that are rate-limited by diffusion at a surface. The average exponent (*γ*) of 0.66 for the uncured VER is in reasonable agreement with this kinetics, suggesting that the changes in electrical resistance obey a rate-limited diffusion process. Additionally, the calculated diffusion coefficient (*D*) of the uncured VER (20.0 × 10^−3^ cm^2^/s) is also in reasonable agreement with the diffusion coefficient (6.3 × 10^−3^ cm^2^/s) of the epoxy vinyl-ester resin used in the studies conducted by Fernández-Toribio et al. [3]. The average exponent (*γ*) for the styrene monomer is 0.63, suggesting that it also obeys this rate-limited diffusion. Furthermore, it is known that the wicking rate depends on the viscosity of the liquid [33], with lower viscosity liquids (such as styrene monomer) presenting a higher wicking rate.

**Figure 8:**
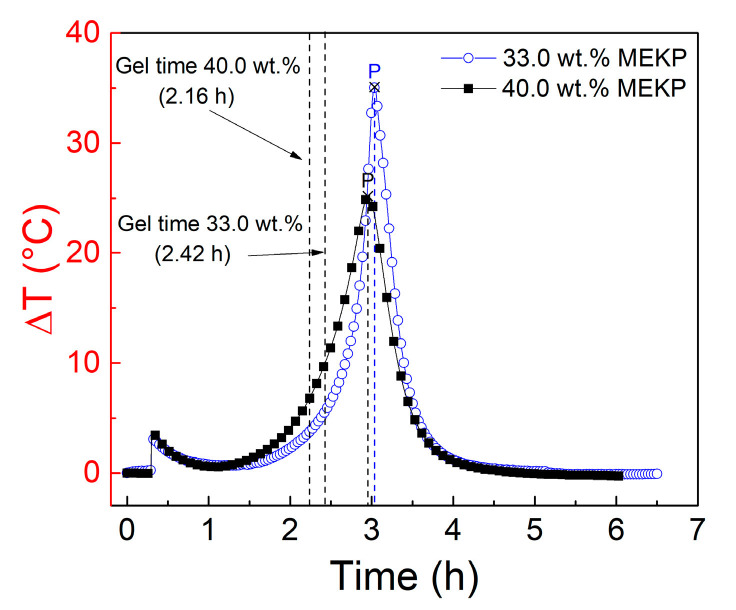
Curing thermograms of 14 mL of VER with 33 and 40 wt.% styrene concentrations.

In order to gain further insight on the effect of the viscosity of the VER resin on the electrical response of the CNTY, additional experiments were conducted by increasing the concentration of styrene contained in the resin. The as-received VER has a 33 wt.% content of styrene monomer, and this is referred to herein as “nominal” styrene content. Additional experiments (three-replicates test plan) were conducted where styrene monomer was added to the VER resin to generate a more dilute (lower viscosity) resin with 40 wt.% styrene content. Electrical measurements were conducted slightly before and during resin curing, such as those described in Section 3.2. Average gel times measured within the monofilament coupon (14 mL), at room temperature (RT ~25 °C), were 2.42 h and 2.16 h for VER resins with 33 wt.% styrene and with 40 wt.% styrene, respectively. In Figure 8, the change in temperature (Δ*T* = *T* − *T*_0_, where *T*_0_ = 25 °C) during curing of the two concentration experiments is plotted as a function of time. Peak exothermic temperatures (point *P*) are identified, and corresponding 14 mL resin volume gel times are included alongside their respective curves. 

**Figure 9:**
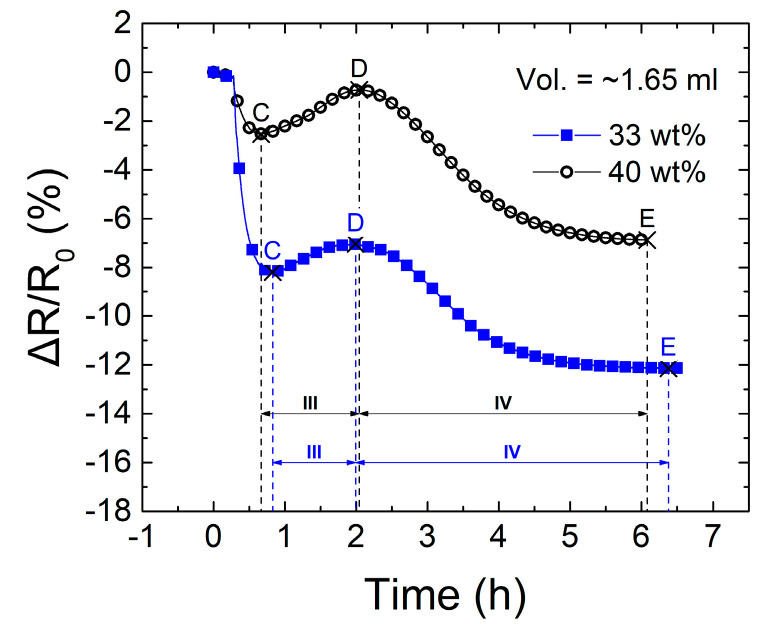
CNTY fractional change of resistance versus time during curing of VER with 33 and 40 wt.% styrene monomer concentrations.

As seen by comparing both curves, increasing the styrene concentration of the VER by 7 wt.% caused a reduction in the gel time and exothermic peak temperature. Figure 9 shows a comparison of the electrical measurements of the single-filament yarn coupons for both styrene monomer concentrations investigated. As pointed out previously, changes in temperature (exotherms) in these experiments (coupons of ~1.65 mL volume, Figure 2) were negligible. The electrical response of the yarn was explained in Section 3.2, by dividing the response curve into four zones, and such a rationale still holds when the styrene content is increased to 40 wt.%. However, for 40 wt.% styrene content, the curve is shifted upwards, and the magnitude of the electrical resistance change diminishes at all points (C through E).

While the nominal viscosity (33 wt.% styrene) curve reaches its local minimum (point C) at ~50 min, the one with reduced viscosity (40 wt.% styrene) does so at ~40 min. A similar situation occurs for points D and E, which occur earlier for the VER resin with 40 wt.% styrene. This is because the additional styrene also modifies the curing kinetics, modifying the chemical VER composition. Interesting conclusions can be drawn from the comparison of Figure 7 and Figure 9. From Figure 7a, notice that the presence of uncured VER yields a monotonic increase of electrical resistance, where *R* never drops. The sole styrene monomer in Figure 7b also yields an increasing trend in *R*, but this is far more rapid and less stable towards the end of the curve, with a small decreasing trend towards the end. The difference between both curves can be rationalized into two major mechanisms, viz. (i) an electrochemical interaction between the yarn and resin/styrene, depleting the states in the conduction band of the yarn and, (ii) wicking and ingress of fluid among bundles/pores of the yarn, which substitutes bundle/air interfaces for resin/air interfaces, where VER and styrene have larger permittivity than air. Notice that, in Figure 7, the effect (i) occurs within a few minutes, and the process becomes fairly stable in ~20 min. Therefore, the fact that both curves in Figure 9 present non-monotonic (upwards and downwards) trends imply that other mechanisms such as chemical shrinkage and development of curing residual stresses should be affecting the electrical response curve of the curing VER. The upward shift of the resistance curve in Figure 9 for the resin with higher concentration of styrene is in accordance with the upward trend observed in Figure 7b, and it is also in agreement with the mechanisms (i) and (ii) described above. 

### 3.4. Scanning Electron Microscopy

**Figure 10:**
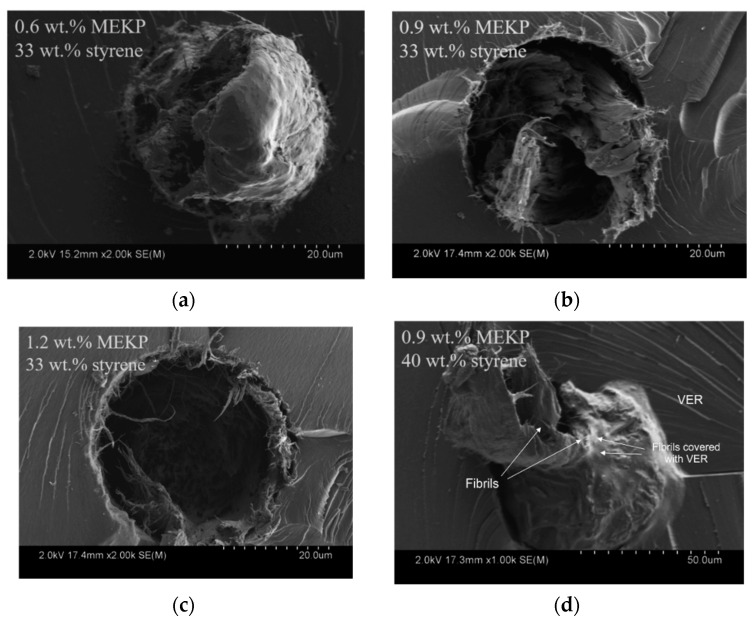
SEM images of solid CNTY/VER monofilament composites taken after deliberate tensile fracture: (**a**) 0.6 wt.% MEKP, (**b**) 0.9 wt.% MEKP, (**c**) 1.2 wt.% MEKP, and (**d**) 0.9 wt.% MEKP and 40 wt.% styrene.

In order to assess the amount of resin infiltrated into the yarn after the end of the curing experiments, SEM analysis was conducted on solid CNTY/VER monofilament composite experiments that were prepared as described in Section 2.4. Figure 10 shows the cross-section of the investigated specimens at 2000×, showing the yarn, interface, and resin. All images (tensile fracture) show an indication of the yarn being pulled out and/or fractured, except the last one, 40 wt.% styrene (Figure 10d), where slipping of bundles/fibrils within the CNTY is more clearly visible. Bundles close to the yarn/resin interface appear slightly more densified than the ones close to the core of the yarn, indicating that wetting and wicking has occurred in an inter-bundle fashion. In Figure 10d, the resin (VER) appears to be covering the fibrils comprising the yarn, as indicated by the arrows within the figure. Thus, for 40 wt.% (Figure 10d), the lower viscosity of the resin causes more wetting of the CNT fibrils comprising the yarn. 

## 4. Conclusions

The electrical response of an individual carbon nanotube yarn (CNTY) embedded in a vinyl ester resin (VER) during polymerization was investigated. It was found that the immersion of individual CNTYs into VER causes abrupt and nonmonotonic changes of electrical resistance that are explained by wetting, wicking, infiltration, electronic transfer, and polymer shrinkage mechanisms, with some of them occurring concurrently. By changing the polymerization kinetics, it was found that the change in concavity of the electrical resistance curve (inflection point) can be correlated with the gel time, determined independently. The amount of free radicals generated from the addition of initiator to the system is a relevant factor that affects not only the polymerization kinetics, but it may also affect electron transfer during the early stages of the resin wicking the yarn. An increase in initiator concentration effectively increased the amount of free radicals, which act as electron donors, increased the rate of monomer conversion, and, in turn, increased the rate of resin viscosity change. Upon wetting the CNTY with VER, a fast transient decrease in the yarn’s electrical resistance was observed for all initiator concentrations. The combination of surface wetting, wicking, and free radicals, as well as doping the yarn’s surface, was credited with this response. This electrochemical phenomenon was characterized by resin filling capillary voids and gaps about the yarn’s irregular surface, followed by infiltration and wicking between intra-bundle voids. As free radicals within the reacting system became depleted due to the progression of the polymerization reaction, the electrical resistance stabilized. The “low” (0.6 wt.%) and “nominal” (0.9 wt.%) initiator concentration were characterized by a nonmonotonic electrical behavior, with a subsequent increase in electrical resistance; this was markedly different from the highest initiator concentration (1.2 wt.%), which gelled quickly and whose resistance decreased faster and monotonically during the full curing experiment. Once the resin gelled, a decrease in electrical resistance is observed, which is attributed to polymer shrinkage, exerting radial compressive stresses to the CNTY. The influence of resin viscosity on infiltration into the CNTY was investigated by varying the styrene concentration of the reaction mixture. An increase in styrene monomer concentration resulted in an upward shift of electrical resistance to less negative values, which was correlated with a higher degree of resin infiltration, as observed by scanning electron microscopy. 

It is thus shown that CNTYs are smart materials whose electrical response during thermosetting resin polymerization may assist in the development of sensory materials for monitoring polymer curing and resin flow, as well as for measuring residual curing stresses.

## Figures and Tables

**Table 1:** Vinyl ester resin (VER) mixtures used to investigate the effect of varying the initiator concentration.

Component	Low Initiator Concentration		Nominal Initiator Concentration		High Initiator Concentration	
	Mass (g)	Concentration (wt.%)	Mass (g)	Concentration (wt.%)	Mass (g)	Concentration (wt.%)
Derakane	29.6	98.8	29.6	98.5	29.5	98.2
CoNap	0.18	0.60	0.18	0.60	0.18	0.60
MEKP	0.18	0.60	0.27	0.90	0.36	1.20

MEKP, methyl ethyl ketone peroxide.

**Table 2:** Masses used in the experiments of modified viscosity (by adding styrene monomer).

Viscosity Label	Derakane (g)	Added Styrene (g)	Total Styrene (g)	Total Styrene (wt.%)
Nominal	29.6	0.0	9.75	33
Low	29.6	3.28	13.0	40

**Table 3:** Gel time and peak exotherm (average and one standard deviation) measured on 14 mL cups for different MEKP concentrations.

Parameter	MEKP Initiator		
	0.6 wt.%	0.9 wt.%	1.2 wt.%
Gel time (h)	6.39 ± 0.06	2.40 ± 0.01	0.98 ± 0.03
Δ*T* peak position (h)	7.81 ± 0.39	3.0 ± 0.06	1.30 ± 0.05
Δ*T* peak exotherm (°C)	2.61 ± 1.32	35.7 ± 4.60	104 ± 2.78

**Table 4:** Summary of zones that characterize the electrical response of the CNTY upon immersion in VER resin.

Zone	Points	Description	Electrical Resistance Change
I	A-B	Dry pre-stressed CNTY within mold.	Small decrease in Δ*R/R*_0_, attributed to yarn stress relaxation.
II	B-C	Liquid VER mixture poured into mold, wetting and wicking into the CNTY.	Transient decrease in Δ*R/R*_0_, attributed to free radical electron donors competing with initial wicking.
III	C-D	Viscosity of the liquid reaction mixture increases to point of gelation.	Increase in Δ*R/R*_0_, attributed to resin wicking and depletion of free radicals due to monomer conversion.
IV	D-E	Gelled VER crosslinks and shrinks around the CNTY.	Decrease in Δ*R/R*_0_, attributed to an increase in contact point density and a decrease in yarn porosity from the radial compressive stresses upon shrinking.

**Table 5:** Viscosities and average fitting parameters of Equation (1) after immersion in uncured VER and styrene monomer.

Immersion Liquid	Dynamic Viscosity (cP) ~25 °C	r^2^	(Δ*R*/*R*_0_)_Eq_ (%)	γ (Exponent Factor)	D (cm^2^/s) ×10^−3^
Uncured VER	325	0.97	4.31	0.66	20.0
Styrene monomer	0.76	0.47	5.41	0.63	195

## Data Availability

The data presented in this study are available upon request from the corresponding author.

## Supplementary Materials

The following are available online, at https://www.mdpi.com/2073-4360/13/5/783/s1. Figure S1: VER thermogram of coupon in Figure 2 (~1.65 mL), using 1.2 wt.% MEKP concentration. Table S1: Summary of ~1.65 mL volume gel time and peak exotherm averages within one standard deviation for different MEKP concentrations.
