# Supplementary material for: Effect of Polymer Viscosity and Polymerization Kinetics on the Electrical Response of Carbon Nanotube Yarn/Vinyl Ester Monofilament Composites
Source: Polymers (Basel). 2021 Mar 4;13(5):783. doi: 10.3390/polym13050783 (PMC7962033; doi:10.3390/polym13050783)
Supplement: Supplementary file 1 [file polymers-13-00783-s001.pdf]

Effect of polymer viscosity and polymerization kinetics on the electrical response of carbon nanotube yarn/vinyl ester monofilament composites

O. Rodríguez-Uicab[1], I. Guay[1], J. L. Abot[1], F. Avilés[2,*]

[1] Department of Mechanical Engineering, The Catholic University of America, Washington, DC 20064, USA.

[2] Centro de Investigación Científica de Yucatán A.C., Unidad de Materiales, Calle 43 No. 130 x 32 y 34, Col. Chuburná de Hidalgo. C.P. 97205, Mérida, Yucatán, Mexico.

Supplementary Information

1. Exothermic curing reaction of the neat vinyl ester resin at reduced resin volume

The results of this section show the curing results for experiments directly measured on the coupon shown in Fig. 2 of the main MS, whose volume is ~ 1.65 ml. Figure S1 shows the results of experiments conducted within the electrical coupon of Fig. 2 at 1.2 wt % MEKP concentration, where significantly smaller changes in temperature than those experienced for higher resin volumes (Fig. 4c) are measured. This is because the peak temperature of the exothermic reaction depends on the volume of the resin [43].

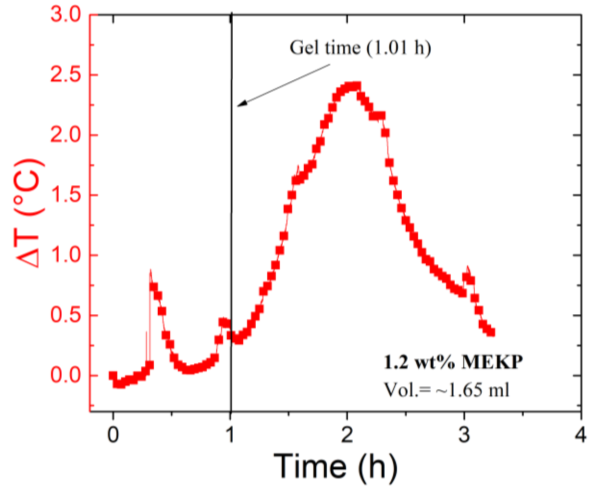

Figure S1. VER thermogram of coupon in Fig. 2 (~1.65 ml) using 1.2 wt.% MEKP concentration.

Table S1 shows the gel times (average and one standard deviation) and peak exothermic temperature. For the experiments using 1.65 ml volumes, the change in temperature for the three

tests ranged from ~ 0.40 °C to ~ 2.68 °C. All gel times preceded peak exothermic temperatures, but occurred during or near the onset of temperature increase.

Table S1. Summary of ~1.65 ml volume gel time and peak exotherm averages within one standard deviation for different MEKP concentrations.

**Parameter**	**MEKP initiator**		
	**0.6 wt.%**	**0.9 wt.%**	**1.2 wt.%**
**Gel time (h)**	6.34±0.06	2.42±0.01	1.01±0.03
***ΔT* peak exotherm (°C)**	0.40±0.36	1.53±0.39	2.68±0.25

References

Reference numbers listed in the Supplementary Information correspond to the list of References included in the main manuscript.
